# Personalized treatment of infertility – way to increase treatment efficiency

**DOI:** 10.1186/1878-5085-5-S1-A151

**Published:** 2014-02-11

**Authors:** Milena Kralickova

**Affiliations:** 1 Biomedical Centre, Faculty of Medicine in Plzen, Charles University in Prague, Plzen, Czech Republic

## Objectives

To overcome the still not sufficient implantation rate following *in vitro* fertilization (IVF) and intracytoplasmatic sperm injection (ICSI) in humans, more than two embryos are commonly replaced, potentially leading to high number of multiple pregnancies with associated significantly elevated risks like low birth weight.

One of the solutions for avoiding multiple pregnancies could be optimalization of embryo selection and single embryo transfer [1]. The traditional systems for assessing gamete and embryo viability have limited ability to accurately select those with best developmental potential. Morphological evaluation remains the primary method of the oocyte and embryo assessment during IVF cycles, but its modest predictive power and inherent inter- and intra-observer variability limits its value. Prolonged embryo culture to blastocyst stage was introduced as a possible strategy for selecting the best embryos, though not even this method brought the solution. The extended period of in-vitro culture may have its side effects – e.g. predispose the embryo to phenomena such as 'large offspring syndrome', which is probably linked to altered gene expression, particularly of imprinted genes.

The unexplained repeated implantation failure (RIF) is still another unsolved and one of the principal problems to affect the outcome of assisted reproductive technology (ART). The successful embryo implantation requires accurate temporal regulation of maternal immune function (to accommodate a semi-allogeneic embryo) and endocrine system [2]. The past few years have witnessed a virtual explosion in the identification of gene mutations or polymorphisms that cause or are linked to human infertility and that are inherited in a polygenic/multifactorial fashion.

## Suggested technologic approaches

The “best viable” embryo selection and also the optimal implantation regulation, both are going to be solved by new diagnostics and treatment methods based on personalized omics profiling [3]. Molecules of embryo-endometrial cross talk that are engaged in regulation of the embryo implantation and selected components of follicular fluid as well as embryo media will play a role on a developmental potential of the embryo and treatment efficiency.

## Expected impact if the project will succeed

The goal of our effort is to point out new and potentially useful biomarkers that can be used for the improvement of pregnancy rates after the assisted reproduction techniques (ART) in near future and also to emphasize some of the novel molecular targets and biomarkers that have been reported recently and might contribute to new strategies of infertility management that will return a hope of natural conception for selected couples.

Evaluation of such markers may help clinicians to predict pregnancy outcome and detect occult implantation deficiency. Moreover, these novel molecular targets are expected to apply to the clinical practice from bench to bedside and improve the implantation efficiency in ART.
